# Development of Colloidal Gold-Based Lateral Flow Immunoassay for Rapid Qualitative and Semi-Quantitative Analysis of Ustiloxins A and B in Rice Samples

**DOI:** 10.3390/toxins9030079

**Published:** 2017-02-24

**Authors:** Xiaoxiang Fu, Rushan Xie, Jian Wang, Xiaojiao Chen, Xiaohan Wang, Weibo Sun, Jiajia Meng, Daowan Lai, Ligang Zhou, Baomin Wang

**Affiliations:** 1 Department of Plant Pathology, College of Plant Protection, China Agricultural University, Beijing 100193, China; xioxiaofu@cau.edu.cn (X.F.); erinxrs@163.com (R.X.); wangjiancau@163.com (J.W.); wangxiaohan99@126.com (X.W.); sunweibo.1001@163.com (W.S.); mengjiajiax@163.com (J.M.); dwlai@cau.edu.cn (D.L.); 2 Department of Crop Physiology and Cultivation, College of Agronomy and Biotechnology, Beijing 100193, China; chenxj@126.com

**Keywords:** ustiloxins A and B, mycotoxin, lateral flow immunoassay, rice false smut disease, *Villosiclava virens*, rice false smut balls, rice grains

## Abstract

Rice false smut is a worldwide devastating rice disease infected by the fungal pathogen *Villosiclava virens*. Ustiloxin A (UA) and ustiloxin B (UB), cyclopeptide mycotoxins, were the major ustiloxins isolated from the rice false smut balls (FSBs) that formed in the pathogen-infected rice spikelets. Based on the specific monoclonal antibodies (mAbs) 2D3G5 and 1B5A10, respectively, against UA and UB, the lateral flow immunoassays (LFIAs) were developed, and the indicator ranges for UA and UB both were 50–100 ng/mL. The cross-reactivities of UB for UA LFIA, and UA for UB LFIA were 5% and 20%, respectively, which were consistent with the icELISA results reported previously. Even at 50,000 ng/mL, none of other commonly existent metabolites in rice samples caused noticeable inhibition. The LFIAs were used for determination of UA and UB contents in rice FSBs and rice grains, and the results were agreeable with those by HPLC and icELISA. There was no change in the sensitivity of either dipstick stored at 4 °C after at least three months. The developed LFIA has specificity and sensitivity for detecting UA and UB as well as simplicity to use. It will be a potential point-of-care device for rapid evaluation of the rice samples contaminated by UA and UB.

## 1. Introduction

**Figure 1:**
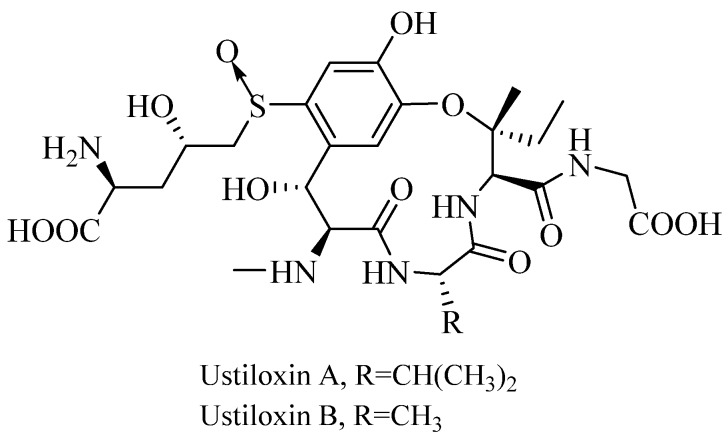
Chemical structures of ustiloxin A (UA) and ustiloxin B (UB).

Rice false smut is a worldwide destructive rice disease caused by the ascomycete fungus *Villosiclava virens* (Nakata) Tanaka and Tanaka (anamorph: *Ustilaginoidea virens* Takahashi) [1] in most rice-growing areas such as China, India, Japan and USA over the past few years [2,3,4,5]. At the early infection stage, the rice false smut ball (FSB) was formed as a white fungal mass protruding from the inner space of a spikelet, followed by transformation into a light-yellow smut ball which, ultimately, changed color to greenish-black after infection of the pathogen into rice filament [6,7]. With the recent widespread cultivation of hybrid rice and heavy application of nitrogenous fertilizer, the rice false smut disease has become significant, resulting in yield loss and grain contamination, and even more importantly, generating mycotoxins (i.e., ustiloxins and ustilaginoidins) that are toxic to plants as well as humans and domestic animals [8,9,10,11,12,13,14]. Ustiloxins, containing a 13-membered cyclic core structure with a phenol ether linkage, were isolated and identified from rice FSBs and named as ustiloxins A, B, C, D, F and G. Among them, ustiloxin A (UA) and ustiloxin B (UB) (Figure 1) are the most toxic and represent more than 80% of the total ustiloxin content [15,16]. It has been reported that ustiloxins had antimitotic activity by inhibiting microtubule assembly and cell skeleton formation of plant and animal cells [17,18,19,20,21]. When domestic animals were fed with the rice grains and feedstuff contaminated by the rice false smut pathogen, they showed a variety of symptoms such as diarrhea, hemorrhage, poor growth, ovarian atrophy, abortion, and damage of liver, heart and kidney [9]. Furthermore, the crude water extract of rice FSBs was found to cause necrosis of the liver and kidney in mice quite similar to that observed in mice lupinosis caused by phomopsin A [22,23]. Therefore, both rice FSBs and false smut pathogen-contaminated rice food and forage have created concerns for food and feed safety.

Several analytical methods for detecting ustiloxins especially for UA and UB have been reported, including high-performance liquid chromatography (HPLC) and liquid chromatography-tandem mass spectrometry (LC-MS/MS) [15,24,25]. Conventional instrumental methods are unsuitable for high-throughput screening of a large number of samples as they are time consuming and labor intensive in addition to the need for expensive instruments. The enzyme-linked immunosorbent assay (ELISA) methods have also been reported for UA and UB [26,27]. However, ELISA method still requires labor-intensive operations, including incubation, washing, enzymatic reactions and instrument analysis.

Recently, the lateral flow immunoassay (LFIA) has become increasingly popular as an efficient screening method for conducting on-site tests with their simplicity, speed, specificity and sensitivity [28,29]. Compared with the conventional ELISA, LFIA is an economic method with a simple sample preparation, and the results can be read by naked eyes within 3–10 min, which is highly suitable as a point-of-care diagnostic device without using expensive instrumentation [30]. However, a convenient and easy-to-use diagnostic device for rapid evaluation of the ustiloxin contamination in rice samples at the point-of-care is still lacking. To develop such a LFIA, the antibody has been considered as the core reagent. Fortunately, two highly specific monoclonal antibodies (mAbs) 2D3G5 and 1B5A10 against UA and UB, respectively have been obtained [26,27]. In the present work, two LFIAs to UA and UB using the specific mAbs 2D3G5 and 1B5A10 were developed, respectively. Their suitability for qualitative and semi-quantitative analysis of UA and UB contents in rice samples were evaluated.

## 2. Results and Discussion

### 2.1. Development of Colloidal Gold-Based LFIA

The sensitivity of the LFIA is based on visual evaluation of the color intensity of the test and control lines. The indicator range was defined as the lowest concentration of the target analyte between which the test line was not visible. In order to obtain the lowest indicator ranges for UA and UB, the concentrations of UA-OVA or UB-BSA conjugate, and goat anti-mouse antibody coated on the membrane and the amounts of colloidal gold-mAb were optimized. The final optimum concentrations of UA-OVA conjugate, goat anti-mouse antibody and colloidal gold-mAb 2D3G5 for UA were 1 mg/mL, 1 mg/mL, 20 µg/mL, respectively. Similarly, the optimized concentration of UB-BSA conjugate, goat anti-mouse antibody and colloidal gold-mAb 1B5A10 were 0.3 mg/mL, 0.5 mg/mL and 20 µg/mL, respectively.

**Figure 2:**
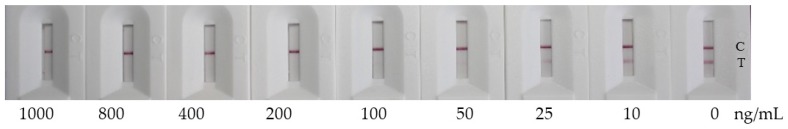
Color changes corresponding to concentrations of UA in distilled water (from left to right: 1000, 800, 400, 200, 100, 50, 25, 10, and 0 ng/mL). The indicator range was 50–100 ng/mL. The letter C represents the control line, while the letter T represents the test line. Each sample dilution was analyzed in triplicate and the figure showed the representative picture.

**Figure 3:**
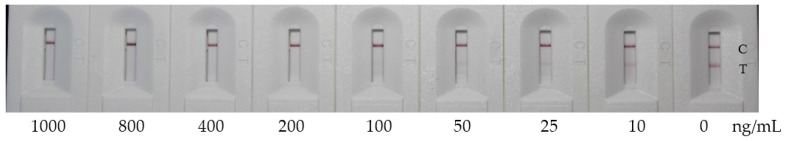
Color changes corresponding to concentrations of UB in distilled water. The indicator range was 50–100 ng/mL. The letter C represents the control line, while the letter T represents the test line. Each sample dilution was analyzed in triplicate and the figure showed the representative picture.

The concentration-dependent color intensity of the LFIAs for mycotoxins UA and UB in distilled water were illustrated in Figure 2 and Figure 3. The indicator range was 50–100 ng/mL both for UA and UB.

To determine the specificities of the LFIAs of UA and UB, the main mycotoxins (UA, UB, ustilaginoidins A, D, E and I) in rice FSBs were used for the evaluation of cross reactivities. For LFIA of UA, the indicator range of UB concentration was 1000–2000 ng/mL (Figure S1A). The cross reactivity to UB was approximately 5%, which agreed with the result by icELISA (4.1%) [26]. No inhibition was observed for ustilaginoidins A, D, E and I respectively at concentration of 50,000 ng/mL, whereas the test line was completely inhibited by UA at 100 ng/mL (Figure S1B), suggesting that this LFIA had no cross reactivity with other mycotoxins (i.e., ustilaginoidins A, D, E and I) in rice FSBs. The cross reactivities against aflatoxin B1 (AFB1), zearalenone (ZEN) and deoxynivalenol (DON) were also tested. But no cross reactivities were observed (Figure S3A).

For LFIA of UB, 250–500 ng/mL of indicator range for UA (Figure S2A) displayed the cross reactivity was approximately 20%, which agreed with the results in icELISA [27]. No inhibition was observed for ustilaginoidins A, D, E and I at concentration of 50,000 ng/mL, whereas the test line was completely inhibited by UB at 100 ng/mL (Figure S2B), suggesting that this LFIA had no cross reactivity with ustilaginoidins in rice FSBs. No cross reactivities were observed against AFB1, ZEN and DON (Figure S3B).

### 2.2. Semi-Quantitative Analysis of UA and UB Contents in Rice FSBs and Grains

The contents of UA and UB in rice FSB and grain samples collected from different regions in China were examined with the LFIAs using several dilutions and compared with the results by HPLC and ELISA. For semi-quantitative analysis, the stock solutions of UA and UB were diluted into 2500-, 5000-, 10,000-, 20,000-, and 40,000-fold to obtain predicted concentration of 400, 200, 100, 50 and 25 ng/mL, respectively.

**Figure 4:**
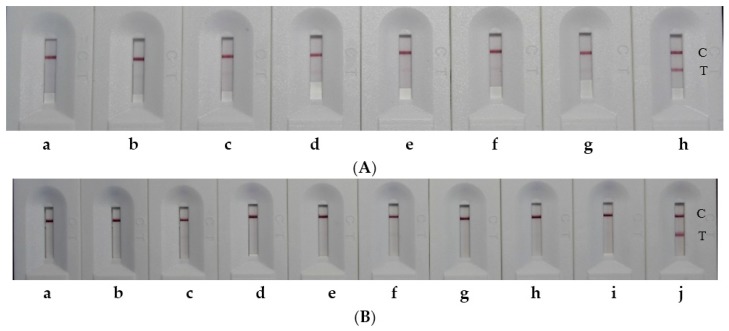
Representative pictures of rice false smut balls (FSBs) tested by lateral flow immunoassay (LFIA). (**A**) LFIA of UA: (**a**) UA (100 ng/mL); (**b**–**g**) represent samples 1, 3, 4, 5, 10 and 19 diluted 1000 fold, respectively; (**h**) water. (**B**) LFIA of UB: (**a**) UB (100 ng/mL); (**b**–**i**) represent samples 1, 3, 4, 5, 7, 10, 13 and 19 diluted 700 fold, respectively; (**j**) water.

The extracts of rice FSB samples were diluted into the appropriate folds with water and tested by LFIA (Figure 4). The UA and UB contents in rice FSBs were also determined by HPLC (Figure S4), and the results agreed well with those of the LFIA (Table 1).

**Figure 5:**
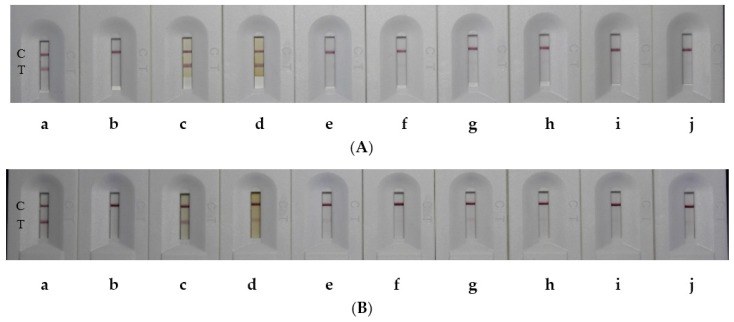
Representative pictures of rice grains tested by LFIA. (**A**) LFIA of UA: (**a**) water; (**b**) UA (100 ng/mL); (**c**) sample 1 without dilution; (**d**) sample 2 without dilution; (**e**) sample 3 diluted 500 fold; (**f**) sample 6 diluted 500 fold; (**g**) sample 17 diluted 500 fold; (**h**) sample 4 diluted 1000 fold; (**i**) sample 5 diluted 1500 fold; (**j**) sample 16 diluted 1500 fold. (**B**) LFIA of UB: (**a**) water; (**b**) UB (100 ng/mL); (**c**) sample 1 without dilution; (**d**) sample 2 without dilution; (**e**) sample 6 diluted 200 fold; (**f**) sample 3 diluted 500 fold; (**g**) sample 17 diluted 500 fold; (**h**) sample 4 diluted 600 fold; (**i**) sample 16 diluted 600 fold; (**j**) sample 5 diluted 1500 fold.

For rice grain samples, the extracted solutions were diluted into the appropriate folds to estimate with LFIA (Figure 5). The UA and UB contents in rice grains were also detected by icELISA and HPLC (Figure S4) and the results agreed well with those of the LFIA (Table 2).

It was not detectable for UA and UB contents in rice grains collected from the rice false smut disease-free regions. While the contents of UA and UB in rice FSBs (0.37–1.27 mg/g for UA; 0.16 to 0.66 mg/g for UB) (Table 1) and contaminated rice grains (1.99 to 107.90 µg/g for UA; 1.04 to 82.57 µg/g for UB) (Table 2) varied and were almost consisted with the results reported previously [26,27]. By analysis, the total content of UA and UB (0.58 to 1.93 mg/g) in rice FSBs was 3–640 fold greater than that (3 to 190 µg/g) in rice grains. In many rice cultivation areas, both people and livestock mainly consume the rice grains contaminated with rice FSBs, which requires attention to food and feed safety. Monitoring contents of ustiloxins in rice grains and their products should be necessary. The developed LFIAs would be useful devices for high throughput screening and rapid evaluation of the rice samples contaminated by UA and UB, or even semi-quantifying UA and UB contents.

### 2.3. Stability of the LFIAs after Storage

The shelf life of the LFIAs was evaluated under three storage conditions (Table S1). There was no change in the sensitivity of either LFIA after a week at 37 °C; three months at 4 °C and ambient temperature (20 ± 5 °C).

For LFIA of UA, the indicator range of UA increased to 100–200 ng/mL after a storage of six months at 4 °C. Although the sensitivity was remained at 50–100 ng/mL, the colors of test and control lines were changed to light after six months at ambient temperature (20 ± 5 °C).

For LFIA of UB, there was no change in the sensitivity (50–100 ng/mL of UB) after a storage of six months at 4 °C. The indicator range decreased to 25–50 ng/mL after six months at ambient temperature (20 ± 5 °C), since the antibody or antigen might be partly invalid to lighten the colors of test and control lines. The results indicated that 4 °C should be the best condition for LFIA storage.

## 3. Conclusions

In this study, the LFIAs for UA and UB were developed based on the mAbs 2D3G5 and 1B5A10 respectively. The indicator ranges for UA and UB both were 50–100 ng/mL. The cross-reactivities of other mycotoxins in rice samples caused not significant interference. The developed LFIAs were then used for determination of UA and UB content in rice FSBs and rice grains, and the results were consistent with those by HPLC and icELISA. At least three months later, there was no change in the sensitivity of either LFIA stored at 4 °C. Overall, the developed LFIA has its specificity and sensitivity for detecting UA and UB, and simplicity to use. It will be a useful point-of-care device for rapid evaluation of the rice samples contaminated by ustiloxins.

## 4. Experimental Section

### 4.1. Chemicals and Reagents

Ustiloxin A (UA) and ustiloxin B (UB) were isolated and purified as described previously [15,31]. Ustilaginoidins A, D, E and I were prepared from the rice false smut balls [13,32]. GBA+ buffer, Sartorius CN140 membranes, Ahlstrom 8964 glass fiber, GL-b0701 sample pad, H5076 absorbent pad, DB-6 PVC backing, A-9 plastic housing and Aluminum foil pouch were bought from Jieyi Biotechnology Co., Ltd. (Shanghai, China).

### 4.2. Materials

The rice FSB samples were collected from different areas of China at different periods of rice false smut disease. The detailed message of the rice FSB samples was shown in Table S2. The samples were extracted with distilled water according to the procedure described previously [15,26,27]. Briefly, 0.05 g of powdered sample was extracted with distilled water for three times (3 × 1.5 mL, 30 min for each time) in an ultrasonic bath at room temperature, followed by centrifugation at 8500× *g* for 10 min. The supernatant extracts were combined and concentrated by a vacuum freeze dryer to dryness, which the residue was dissolved in 0.5 mL of ultrapure water in a test tube. The extracted solution was divided into two aliquots.

The rice grain samples, infected by *Villosiclava virens* inordinately, were collected from different areas of China. The detailed message of the rice grain samples was shown in Table S3. The samples were extracted with distilled water according to the procedure described previously [15,26,27]. Briefly, 1 g of powdered sample was extracted with distilled water for three times (3 × 6 mL, 30 min for each time) in an ultrasonic bath at room temperature, followed by centrifugation at 8500× *g* for 10 min. The supernatant extracts were combined and concentrated by a vacuum freeze dryer to dryness, which the residue was dissolved in 1 mL of ultrapure water in a test tube. The extracted solution was divided into two aliquots.

### 4.3. Production of mAbs against UA and UB

UA and UB were conjugated to bovine serum albumin (BSA) and ovalbumin (OVA) to prepare the immunogen (UA-BSA, UB-OVA) and coating antigen (UA-OVA, UB-BSA), respectively, using the glutaraldehyde method [26]. The protocol for icELISA was the same as that described previously [26]. The monoclonal antibodies against UA and UB were produced by the positive hybridomas, 2D3G5 and 1B5A10, respectively, as previously reported [26,27]. The cross-reactivity of UB was 4.1% for UA icELISA [26]. The cross-reactivity of UA was 13.9% for UB icELISA [27]. The developed monoclonal antibodies were considered to be specifically used to determine UA or UB content. Therefore, the mAb 2D3G5 and 1B5A10 were selected to develop colloidal gold-based LFIAs for UA and UB. Both 2D3G5 and 1B5A10 were dialysis with PBS-PBS-PBS-PB-PB-H_2_O (PBS: 0.01 M phosphate buffer containing 0.9% NaCl, pH 7.5; PB: 0.01 M phosphate buffer, pH 7.0) and used for LFIA development [26,27].

### 4.4. Development of Colloidal Gold-based LFIA

Using the glutaraldehyde method, UA and UB were conjugated to ovalbumin (UA-OVA) and bovine serum albumin (UB-BSA) to prepare the test capture reagent, respectively [25]. Briefly, 1 mg of UA or UB was dissolved in DMF and then added into 2 mL of PBS containing OVA (7 mg) or BSA (10 mg) while stirring, followed by addition of 6.5 µL of 5% glutaraldehyde solution into the mixture. The reaction mixture was stirred overnight at 4 °C, later dialyzed against PBS.

#### 4.4.1. LFIA of UA

Colloidal gold with an average particle diameter of 30 nm (G30) was prepared according to the procedure previously described [33]. The protocol for LFIA was almost the same as previously described with minor optimization [34,35]. While gentle stirring, 20 μL of aqueous solution of the mAb (1 mg/mL) was added to 1 mL of colloidal gold solution which was adjusted to pH 7.8 with potassium carbonate solution. After coating for 10 min at ambient temperature, 40 μL of 10% (*w*/*v*) BSA was added to the gold-antibody suspension for further stabilizing. The mixture was gently stirred for 15 min, followed by centrifugation at 8500× *g* for 15 min at 4 °C. The supernatant was then carefully discarded, and the pellet was resuspended in 500 μL of 0.01 M PB (pH 7.0). Finally, the conjugate pad was saturated with the gold-antibody conjugate, and dried at room temperature overnight.

For the preparation of the nitrocellulose (NC) membrane, Goat anti-mouse IgG were used as a control capture, and UA-OVA was used as a test capture reagent, respectively. Using a dispenser, both the control and test capture reagents (1 mg/mL) with the volume of 1 μL/cm were immobilized to NC membrane separately as lines. The distance between the two lines was 5 mm. After dispensing, the membrane was dried at room temperature for 60–120 min to immobilize the reagents.

**Figure 6:**
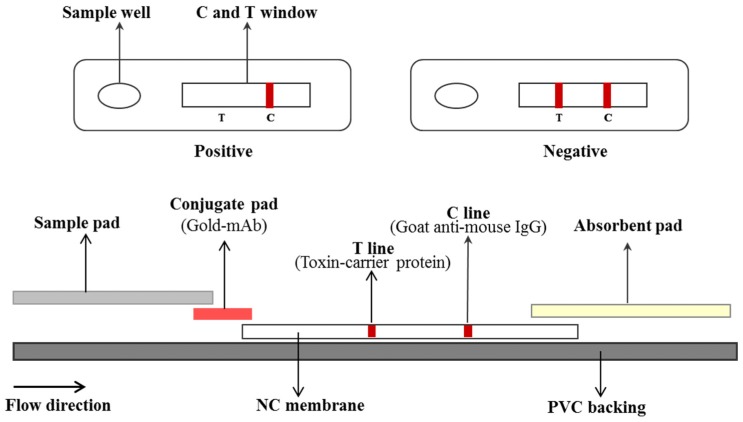
The scheme of the competitive LFIA.

The dipstick was assembled as described previously [35]. The competitive LFIA was consisted of a polyvinyl chloride (PVC), NC membrane, conjugate pad, sample pad and absorbent pad (Figure 6). The NC membrane was pasted onto the center of the PVC plate which was served as the backing of the test strip. The colloidal gold-mAb conjugate pad was pasted with 1–2 mm overlapping with the NC membrane. The absorbent pad was attached to the top of the plate in a similar manner as the conjugate and sample pads. After complete assembly, the card was cut to 3 mm width. Strips were then sealed in a plastic case with desiccant gel.

#### 4.4.2. LFIA of UB

The procedure of colloidal gold-based LFIA for UB test was almost the same as described above (4.3.1) except for some detail parameters. Briefly, the gold-mAb pellet was resuspended in 2000 μL of GBA+ buffer as its slightly inferior solubility in PB. The concentration of control capture reagent (goat anti-mouse IgG) was 0.5 mg/mL, while the test capture reagent (UB-BSA) was 0.3 mg/mL.

### 4.5. Evaluation of the LFIAs: Indicator Range and Cross Reactivity

UA and UB were separately dissolved in sterile water at 1 mg/mL as stock solution. The stock solutions of ustilaginoidins A, I, D and E at 2 mg/mL were prepared in acetone.

To determine the specificity of the LFIA for UA or UB, other common mycotoxins in rice false smut balls were used for the evaluation of cross reactivity. As UA and UB were the predominant ustiloxins in rice FSBs [15,16], and ustilaginoidins A, D, E and I were main ustilaginoidins in rice FSBs [13,36], thus, UA and UB as well as ustilaginoidins A, D, I and E were chosen to detect the cross reactivities of LFIAs. We also tested the cross reactivities against AFB1, ZEN and DON at concentration of 50,000 ng/mL, as they were the major mycotoxins contaminating grains. Cross reactivity (CR) of analyte was calculated refer to the formula used in ELISA: *CR* (%) = (Indicator range (other mycotoxin)/Indicator range (analyte)) × 100.

#### 4.5.1. LFIA of UA

The indicator range, indicated the sensitivity of the LFIA, was defined as the lowest concentration of the target analytes between which the test line was not visible. To estimate the indicator range of the LFIA, the UA standard solution was diluted to 1000, 800, 400, 200, 100, 50, 25, 10, 0 ng/mL with distilled water. Then 80 μL of standard solutions were added dropwise into the sample well. The color of the test and control line was visually observed within 5–10 min.

The UB standard solution was diluted to 20000, 10000, 5000, 2000, 1000, 500, 250, 100, 0 ng/mL with distilled water. Other common mycotoxins (i.e., ustilaginoidins A, I, D and E) in rice FSBs were diluted to 50,000 ng/mL with distilled water. All of the diluted solutions were tested by LFIA for cross reactivity evaluation.

#### 4.5.2. LFIA of UB

The evaluation of the LFIA indicator range and cross reactivity for UB was almost the same as described above (4.4.1) except for some detail parameters. The UB standard solution was diluted to 1000, 800, 400, 200, 100, 50, 25, 10, 0 ng/mL with distilled water to estimate the indicator range.

The UA standard solution was diluted to 5000, 2000, 1000, 500, 250, 125, 50, 25, 0 ng/mL with distilled water to estimate the cross reactivity. The evaluation of cross reactivity for other common mycotoxins (ustilaginoidins A, I, D and E) was the same as 2D3G5 described above.

### 4.6. Comparison of UA and UB Contents in Rice FSBs Tested by LFIA and HPLC

A volume of 100 µL of the extracted solution was diluted into proper folds with distilled water. Then, 80 µL of diluted solution was added dropwise into the sample well. The color of the test and control line was visually observed within 5–10 min. Each sample was detected by LFIA in triplicate.

A volume of 400 µL of the solution with the proper concentration was filtered through a filter (pore size, 0.22 μm) and analyzed on a Shimadzu Prominence LC-20A high-performance liquid chromatography system (Kyoto, Japan) that consisted of two LC-20AT solvent delivery units, an SIL-20A autosampler, an SPD-M20A photodiode array detector, a CBM-20Alite system controller, and a Synergi reversed-phase Hydro-C18 column (250 mm × 4.6 mm, 5 μm) (Phenomenex, Torrance, CA, USA). The injection volume was 50 μL. The mobile phase, composed of methanol-water (15:85, *v*/*v*) containing 0.02% TFA (*v*/*v*), was set at a flow rate of 1.0 mL/min, in isocratic elution mode at a temperature of 40 °C. The detection wavelength was at 220 nm. A total analysis time was 25 min [15]. Each sample was detected by HPLC in triplicate.

### 4.7. Comparison of UA and UB Contents in Rice Grains Tested by LFIA, ELISA and HPLC

A volume of 500 µL of the extracted solution was diluted proper folds with distilled water. Then, 80 µL of diluted solution was added in drops into the sample well. The color of test and control line was visually observed within 5–10 min. Each sample was detected by LFIA in triplicate.

Since the content of UA and UB in most rice grain samples was too low for HPLC assay, the extracted solutions were also determined by icELISA developed previously [26,27]. Each sample was detected in triplicate.

The other aliquot (500 µL) of concentrated solution was then filtered through a filter (pore size, 0.22 μm) and analyzed by HPLC the same as above. Each sample was tested in triplicate.

### 4.8. Stability Test of the LFIAs

To determine the performance of the LFIAs after storage, the dipsticks were stored for one week, one month, 3 months and 6 months at 4 °C, ambient temperature (20 ± 5 °C) and 37 °C. After storage, their indicator ranges for UA or UB were re-evaluated.

## Figures and Tables

**Table 1:** Comparison of UA and UB contents in rice FSBs tested by LFIA and high-performance liquid chromatography (HPLC).

Sample No.	UA Content (mg/g)		UB Content (mg/g)		UA Plus UB Content (mg/g)	
	LFIA	HPLC	LFIA	HPLC	LFIA	HPLC
1	0.5–1.0	0.81 ± 0.03	0.35–0.7	0.36 ± 0.01	0.85–1.7	1.16 ± 0.04
2	0.4–0.8	0.62 ± 0.01	0.25–0.5	0.28 ± 0.02	0.65–1.3	0.91 ± 0.02
3	0.5–1.0	0.88 ± 0.03	0.35–0.7	0.37 ± 0.02	0.85–1.7	1.25 ± 0.07
4	0.5–1.0	0.81 ± 0.01	0.35–0.7	0.33 ± 0.01	0.85–1.7	1.13 ± 0.02
5	0.5–1.0	0.81 ± 0.005	0.35–0.7	0.38 ± 0.02	0.85–1.7	1.19 ± 0.01
6	0.4–0.8	0.57 ± 0.02	0.35–0.7	0.43 ± 0.02	0.75–1.5	0.99 ± 0.04
7	0.4–0.8	0.59 ± 0.01	0.35–0.7	0.32 ± 0.004	0.75–1.5	0.92 ± 0.01
8	0.4–0.8	0.66 ± 0.02	0.25–0.5	0.25 ± 0.01	0.65–1.3	0.90 ± 0.03
9	0.75–1.5	1.04 ± 0.03	0.6–1.2	0.46 ± 0.03	1.35–2.7	1.50 ± 0.04
10	0.5–1.0	0.76 ± 0.05	0.35–0.7	0.37 ± 0.01	0.85–1.7	1.13 ± 0.06
11	0.3–0.6	0.42 ± 0.02	0.2–0.4	0.16 ± 0.003	0.5–1.0	0.58 ± 0.03
12	0.75–1.5	1.27 ± 0.05	0.6–1.2	0.66 ± 0.01	1.35–2.7	1.93 ± 0.06
13	0.75–1.5	0.85 ± 0.03	0.35–0.7	0.36 ± 0.03	1.1–2.2	1.21 ± 0.05
14	0.75–1.5	1.05 ± 0.01	0.6–1.2	0.50 ± 0.01	1.35–2.7	1.55 ± 0.01
15	0.3–0.6	0.37 ± 0.005	0.25–0.5	0.21 ± 0.01	0.55–1.1	0.59 ± 0.01
16	0.3–0.6	0.40 ± 0.03	0.25–0.5	0.19 ± 0.03	0.55–1.1	0.59 ± 0.01
17	0.3–0.6	0.51 ± 0.02	0.25–0.5	0.20 ± 0.02	0.55–1.1	0.71 ± 0.04
18	0.4–0.8	0.59 ± 0.04	0.25–0.5	0.20 ± 0.02	0.65–1.3	0.79 ± 0.06
19	0.5–1.0	0.76 ± 0.02	0.35–0.7	0.23 ± 0.02	0.85–1.7	0.99 ± 0.002

Note: The data analyzed by HPLC are expressed as means of triplicate ± standard deviations (*n* = 3).

**Table 2:** Comparison of UA and UB contents in rice grains tested by LFIA, ELISA and HPLC.

Sample No.	UA Content (mg/g)			UB Content (mg/g)			UA Plus UB Content (mg/g)		
	LFIA	ELISA	HPLC	LFIA	ELISA	HPLC	LFIA	ELISA	HPLC
1	-	-	-	-	-	-	-	-	-
2	-	-	-	-	-	-	-	-	-
3	25–50	33.05 ± 3.20	38.76 ± 1.85	25–50	27.55 ± 1.76	25.52 ± 3.82	50–100	60.60 ± 2.04	64.27 ± 5.67
4	50–100	49.72 ± 2.66	59.16 ± 1.93	30–60	39.31 ± 2.76	29.31 ± 2.80	80–160	89.03 ± 3.26	88.47 ± 4.02
5	75–150	103.19 ± 5.61	107.90 ± 1.36	75–150	96.64 ± 9.60	82.57 ± 7.56	150–300	199.84 ± 4.22	190.47 ± 7.70
6	25–50	21.27 ± 2.28	26.37 ± 3.16	10–20	8.34 ± 0.23	-	35–70	29.61 ± 2.47	-
7	4–8	3.91 ± 0.33	-	2.5–5	2.08 ± 0.18	-	6.5–13	5.98 ± 0.49	-
8	2–4	1.99 ± 0.11	-	1–2	1.04 ± 0.08	-	3–6	3.04 ± 0.10	-
9	2–4	2.44 ± 0.38	-	1–2	1.29 ± 0.10	-	3–6	3.72 ± 0.47	-
10	2–4	2.01 ± 0.14	-	1–2	1.69 ± 0.17	-	3–6	3.70 ± 0.05	-
11	2–4	3.05 ± 0.29	-	1.5–3	1.98 ± 0.15	-	3.5–7	5.03 ± 0.14	-
12	4–8	4.14 ± 0.18	-	2.5–5	2.50 ± 0.44	-	6.5–13	6.64 ± 0.26	-
13	4–8	5.58 ± 0.62	-	2.5–5	3.77 ± 0.16	-	6.5–13	9.35 ± 0.64	-
14	4–8	4.28 ± 0.32	-	2.5–5	2.58 ± 0.10	-	6.5–13	6.86 ± 0.38	-
15	75–150	93.57 ± 8.77	98.89 ± 2.23	30–60	42.64 ± 3.16	37.38 ± 0.81	105–210	136.21 ± 5.78	136.27 ± 1.50
16	25–50	30.86 ± 1.79	40.52 ± 4.69	25–50	23.31 ± 1.73	22.86 ± 2.44	50–100	54.17 ± 3.51	63.38 ± 5.23
17	2–4	3.09 ± 0.16	-	1.5–3	2.20 ± 0.32	-	3.5–7	5.29 ± 0.42	-

Note: The data analyzed by ELISA and HPLC are expressed as means of triplicate ± standard deviations (*n* = 3).

## Supplementary Materials

The following are available online at www.mdpi.com/2072-6651/9/3/79/s1. Table S1: The storage stability of the dipsticks, Table S2: The details of rice FSB samples, Table S3: The details of rice grain samples, Figure S1: Specificity test for LFIA of UA, Figure S2: Specificity test for LFIA of UB, Figure S3: Specificity tests for LFIAs of UA and UB, Figure S4: HPLC chromatograms of ustiloxin A/B as well as the extracts of rice FSBs spiked or unspiked with ustiloxin A/B.
