# Supplementary material for: Development of Colloidal Gold-Based Lateral Flow Immunoassay for Rapid Qualitative and Semi-Quantitative Analysis of Ustiloxins A and B in Rice Samples
Source: Toxins (Basel). 2017 Feb 24;9(3):79. doi: 10.3390/toxins9030079 (PMC5371834; doi:10.3390/toxins9030079)
Supplement: Supplementary file 1 [file toxins-09-00079-s001.pdf]

Supplementary Materials: Development of Colloidal Gold-based Lateral Flow Immunoassay for Rapid Qualitative and Semi-quantitative Analysis of Ustiloxins A and B in Rice Samples

Xiaoxiang Fu, Rushan Xie, Jian Wang, Xiaojiao Chen, Xiaohan Wang, Weibo Sun, Jiajia Meng, Daowan Lai, Ligang Zhou and Baomin Wang

Table S1. The storage stability of the dipsticks.

Analyte	Storage Condition	Indicator Range (ng/mL)				
		1 Day	1 Week	1 Month	3 Months	6 Months
UA (2D3G5)	4 °C	50–100	50–100	50–100	50–100	100–200
	Ambient temperature	50–100	50–100	50–100	50–100	50–100
	37 °C	50–100	50–100	-	-	-
UB (1B5A10)	4 °C	50–100	50–100	50–100	50–100	50–100
	Ambient temperature	50–100	50–100	50–100	50–100	25–50
	37 °C	50–100	50–100	-	-	-

Note: The ambient temperature was 20 ± 5 °C.

Table S2. The details of rice FSB samples.

Sample No.	Collection Area (Longitude and Latitude)	Collection Time
1	Hefei (117.2°E, 31.8°N), Anhui, China	Oct. 2014
2	Hefei (117.2°E, 31.8°N), Anhui, China	Oct. 2014
3	Fengyang (117.5°E, 32.9°N), Anhui, China	Oct. 2014
4	Fengyang (117.5°E, 32.9°N), Anhui, China	Oct. 2014
5	Qianshan (116.6°E, 30.6°N), Anhui, China	Aug. 2012
6	Jianou (118.3°E, 27.0°N), Fujian, China	Nov. 2012
7	Jianyang (118.1°E, 27.3°N), Fujian, China	Nov. 2012
8	Xing'an (110.7°E, 25.6°N), Guangxi, China	Oct. 2015
9	Changsha (112.9°E, 28.2°N), Hunan, China	Nov. 2011
10	Hanshou (112.0°E, 28.9°N), Hunan, China	Oct. 2013
11	Hanshou (112.0°E, 28.9°N), Hunan, China	Sept. 2015
12	Linyi (118.4°E, 35.1°N), Shandong, China	Oct. 2011
13	Linyi (118.4°E, 35.1°N), Shandong, China	Oct. 2012
14	Linyi (118.4°E, 35.1°N), Shandong, China	Oct. 2013
15	Donggang (124.2°E, 39.9°N), Liaoning, China	Oct. 2010
16	Donggang (124.2°E, 39.9°N), Liaoning, China	Dec. 2011
17	Qionglai (103.5°E, 30.4°N), Sichuan, China	Sep. 2012
18	Chengdu (104.1°E, 30.6°N), Sichuan, China	Sep. 2014
19	Zhangjiagang (120.6°E, 31.9°N), Jiangsu, China	Nov. 2015

Note: The collected rice FSB samples were kept at −20°C before use.

Table S3. The details of rice grain samples.

Sample No.	Rice Cultivar	Collection Area (Longitude and Latitude)	Collection Time
1	Zhonghua 17	Shangzhuang (116.2°E, 40.1°N), Beijing, China	Oct. 2013
2	Lijiang	Shangzhuang (116.2°E, 40.1°N), Beijing, China	Oct. 2011
3	H329	Donggang (124.2°E, 39.9°N), Liaoning, China	Nov. 2014
4	H597	Donggang (124.2°E, 39.9°N), Liaoning, China	Nov. 2014
5	Yanfeng 47	Donggang (124.2°E, 39.9°N), Liaoning, China	Nov. 2014
6	Maisui 1	Donggang (124.2°E, 39.9°N), Liaoning, China	Nov. 2014
7	Xiangjing	Donggang (124.2°E, 39.9°N), Liaoning, China	Nov. 2014
8	Liaojing 212-14	Donggang (124.2°E, 39.9°N), Liaoning, China	Nov. 2014
9	Liaokai 79	Donggang (124.2°E, 39.9°N), Liaoning, China	Nov. 2014
10	Yanjing 218	Donggang (124.2°E, 39.9°N), Liaoning, China	Nov. 2014
11	Tianyouhuazhan	Hanshou (112.0°E, 28.9°N), Hunan, China	Oct. 2013
12	Huiliangyou 2000	Hanshou (112.0°E, 28.9°N), Hunan, China	Sep. 2015
13	Shenliangyou 116	Hanshou (112.0°E, 28.9°N), Hunan, China	Sep. 2015
14	Longliangyou 534 1	Hanshou (112.0°E, 28.9°N), Hunan, China	Sep. 2015
15	Shenliangyou 1	Hanshou (112.0°E, 28.9°N), Hunan, China	Sep. 2015
16	Longliangyou 534 2	Hanshou (112.0°E, 28.9°N), Hunan, China	Sep. 2015
17	Shuidao 555	Suzhou (120.6°E, 31.3°N), Jiangsu, China	Nov. 2015

Note: The collected rice grain samples were kept at -20°C before use.

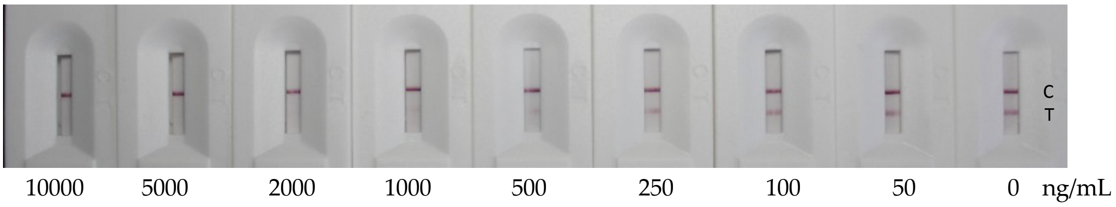

(**A**)

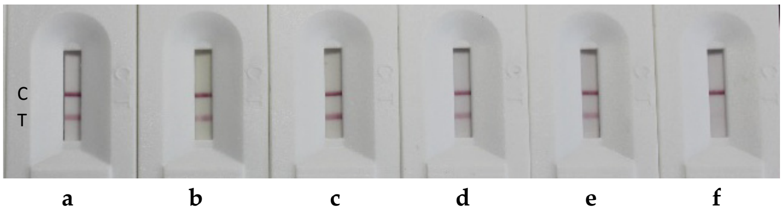

(**B**)

Figure S1. Specificity test for LFIA of UA. (**A**) the indicator range of UB was 1000–2000 ng/mL. (**B**) common mycotoxins in rice FSBs: (**a**) ustilaginoidin I; (**b**) ustilaginoidin A; (**c**) ustilaginoidin D; (**d**) ustilaginoidin E respectively at concentration of 50,000 ng/mL. (**e**) blank; (**f**) UA at 100 ng/mL. The letter C represents the control line, while the letter T represents the test line. Each sample dilution was analyzed in triplicate and the figure showed the representative picture.

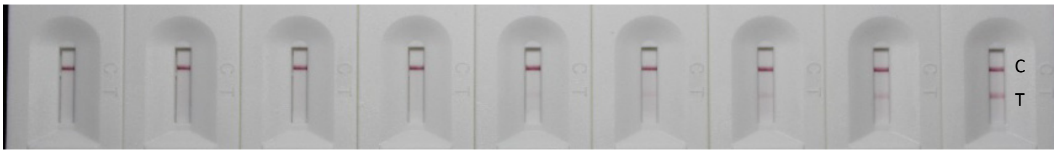

5000 **2000** **1000** **500** **250** **125** **50** **25** **0** ng/mL

(**A**)

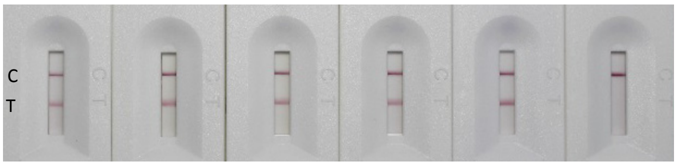

a **b** **c** **d** **e** **f**

(**B**)

Figure S2. Specificity test for LFIA of UB. (**A**) the indicator range of UA was 250–500 ng/mL. (**B**) common mycotoxins in rice FSBs: (**a**) ustilaginoidin I; (**b**) ustilaginoidin A; (**c**) ustilaginoidin D; (**d**) ustilaginoidin E respectively at concentration of 50,000 ng/mL. (**e**) blank; (**f**) UA at 100 ng/mL. The letter C represents the control line, while the letter T represents the test line. Each sample dilution was analyzed in triplicate and the figure showed the representative picture.

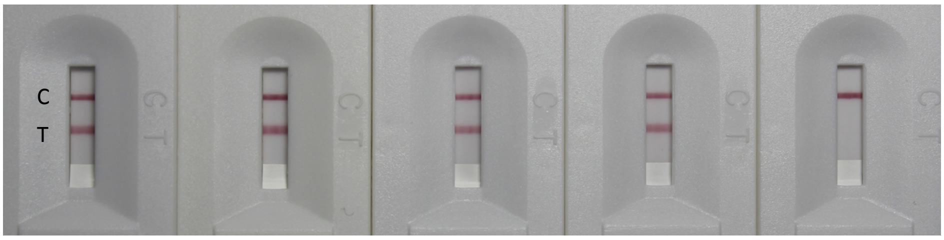

a **b** **c** **d** **e**

(**A**)

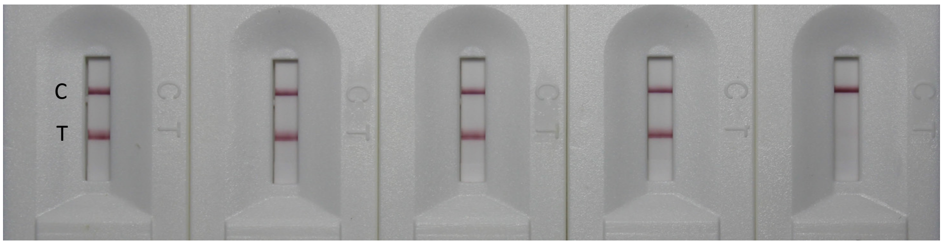

a **b** **c** **d** **f**

(**B**)

Figure S3. Specificity tests for LFIAs of UA and UB. (**A**) Specificity test for LFIA of UA; (**B**) Specificity test for LFIA of UB. (**a**) blank; (**b**) AFB1; (**c**) ZEN; (**d**) DON, respectively at concentration of 50,000 ng/mL; (**e**) UA at 100 ng/mL; (**f**) UB at 100 ng/mL. C represents the control line, while T represents the test line. Each sample dilution was analyzed in triplicate and the figure showed the representative pictures.

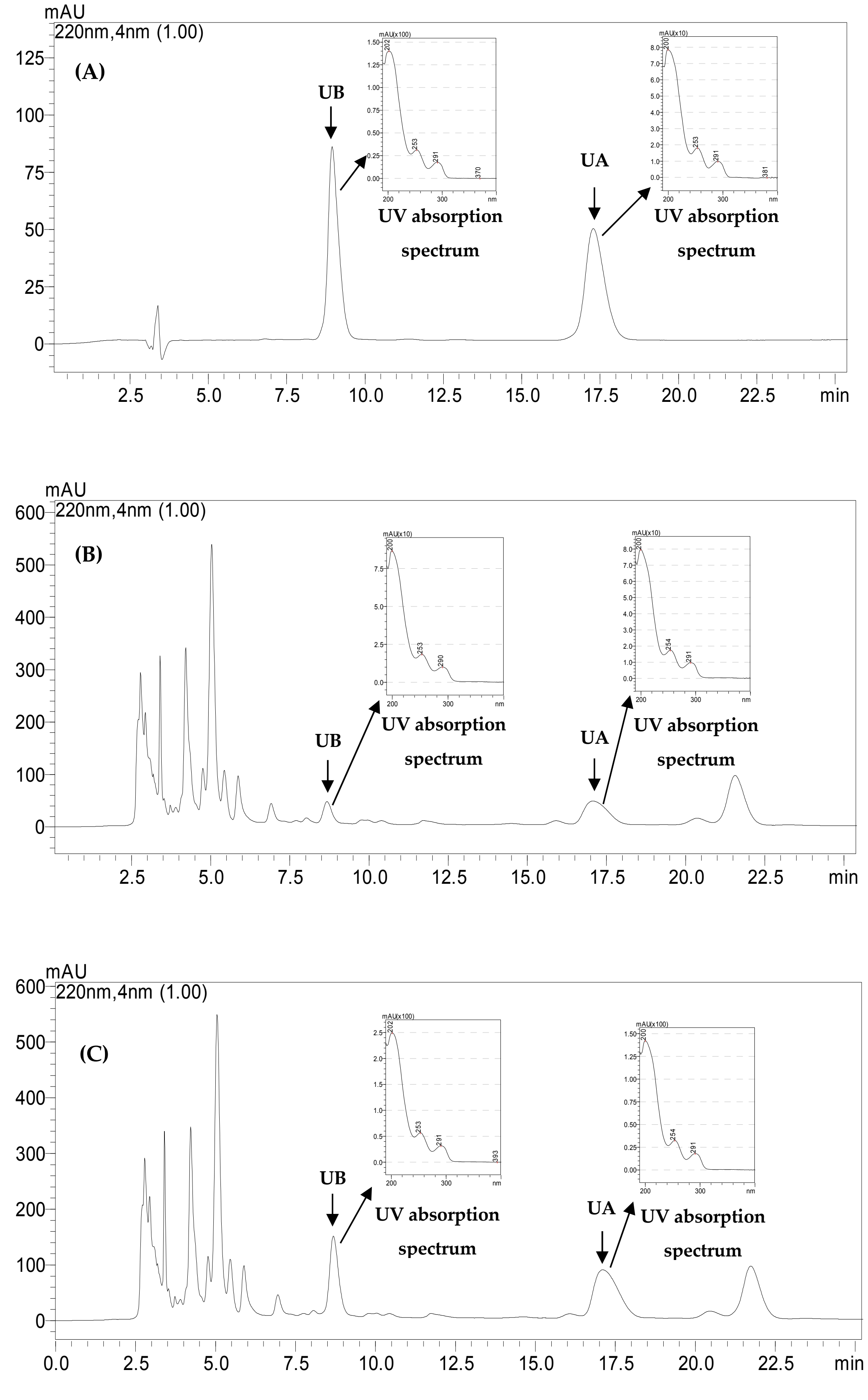
mAU
220nm,4nm (1.00)
(A)
UB
UA
UV absorption spectrum
UV absorption spectrum
min
(B)
UB
UA
UV absorption spectrum
UV absorption spectrum
(C)
UB
UA
UV absorption spectrum
UV absorption spectrum

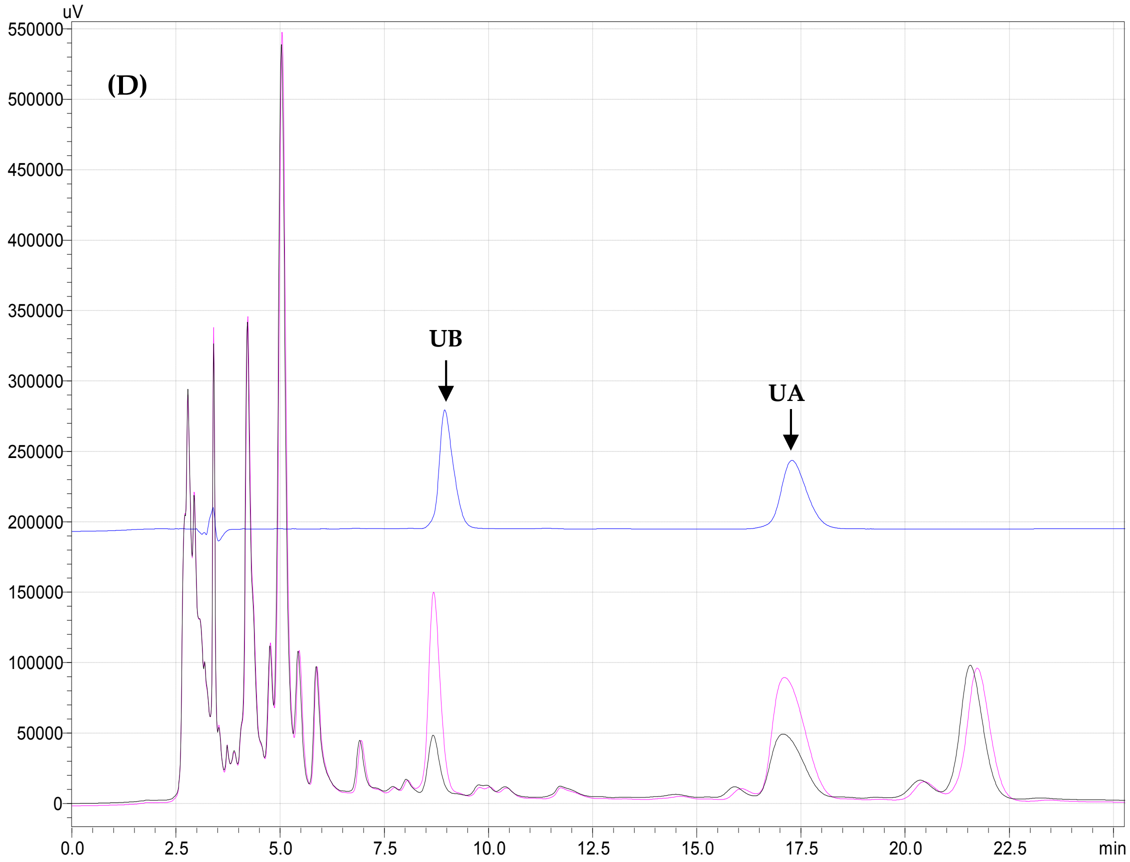

Figure S4. HPLC chromatograms of ustiloxin A/B as well as the extracts of rice FSBs spiked or unspiked with ustiloxin A/B. (**A**) HPLC profile and UV absorption spectra of the authentic ustiloxin A/B (UA/UB); (**B**) HPLC profile and UV absorption spectra of the water extract of rice FSB sample 1 collected from Hefei of Anhui (refer to Table S2); (**C**) HPLC profile and UV spectra of water extract of rice FSB sample 1 spiked with UA and UB; (**D**) Comparison of HPLC profiles of the standard compound solution (——), the water extract of rice FSB sample 1 (——), and the water extract of rice FSB sample 1 spiked with UA and UB (——).
